# Coenzyme Q10-Polyethylene Glycol Monostearate Nanoparticles: An Injectable Water-Soluble Formulation

**DOI:** 10.3390/antiox9010086

**Published:** 2020-01-19

**Authors:** Kengo Banshoya, Tetsuya Nakamura, Tetsuro Tanaka, Yoshiharu Kaneo

**Affiliations:** Faculty of Pharmacy and Pharmaceutical Sciences, Fukuyama University, Gakuen-cho 1, Fukuyama, Hiroshima 729-0292, Japan; kban@fukuyama-u.ac.jp (K.B.); t-nakamura@fukuyama-u.ac.jp (T.N.); tanaka@fukuyama-u.ac.jp (T.T.)

**Keywords:** coenzyme Q10, polyethylene glycol monostearate, water-soluble, micellar formulation, oxidative stress

## Abstract

Therapeutic applications of coenzyme Q10 (CoQ10) are greatly limited by its lack of solubility in aqueous media. In this study, polyethylene glycol monostearate (stPEG) was used to construct micelles containing CoQ10 as a new formulation. The micellar formulations (stPEG/CoQ10) were prepared using five types of stPEG with 10, 25, 40, 55, and 140 PEG repeat units, respectively. The micellar preparation was simple, consisting of only stPEG and CoQ10. Next, we compared the physical properties and blood circulation of these micelles. The CoQ10 load of this formulation was approximately 15 *w/w*%. Based on the dynamic light scattering method, the average molecular size of the stPEG/CoQ10 micelles was approximately 15 to 60 nm. The zeta potentials of these micelles were approximately −10 to −25 mV. The micelles using stPEG25, 40, and 55 demonstrated high solubility in water. Furthermore, these micelles had in vitro antioxidant activity. On comparing the blood circulation of micelles using stPEG25, 40, 55, and 140, micelles using stPEG55 had a significantly higher circulation in blood. The stPEG55/CoQ10 micelle demonstrated a protective effect against acetaminophen-induced liver injury in mice. In conclusion, these data indicate that the intravenous administration of the stPEG/CoQ10 micellar aqueous formulation is of great value against oxidant stress.

## 1. Introduction

Coenzyme Q10 (CoQ10) has been reported to have a wide range of positive therapeutic effects on human health [1]. It is a powerful endogenous antioxidant, reducing the potential negative effects of free radicals [2,3]. As a therapeutic agent, CoQ10 has generated interest in several diseases including mitochondrial diseases, cancer, and cardiovascular disease [4,5,6]. Its structure consists of a benzoquinone ring prenylated with an isoprenoid chain. Because of the long side chain of the molecule consisting of 10 isoprenoid units, CoQ10 is extremely lipophilic and practically insoluble in water. Furthermore, the oral bioavailability of CoQ10 is generally very low and was related to the dissolution rate of the formulation [7]. Hence, intravenous administration systems are particularly important because of this low oral bioavailability.

One approach to improve a hydrophobic drug’s performance involves the use of a polymer micellar system [8,9,10,11]. Polymer micelles formed using amphiphilic block polymers have many advantages such as improved drug solubilization, ease of preparation and storage, stability in body fluids, and long retention times. However, when these micelles are administered to a living organism and enter the bloodstream, they are diluted to a concentration below the critical micelle concentration (CMC), resulting in micelle destabilization. This problem has been resolved by using polyethylene glycol (PEG) as the hydrophilic group [12].

Polyethylene glycol monostearate (stPEG) is mainly used as an emulsifier in topical pharmaceutical formulations; it is also used in intravenous injections and oral formulations. This amphiphilic block polymer has been extensively evaluated for toxicity in animals and is widely used in pharmaceutical formulations and cosmetics, generally regarded as an essentially nontoxic and nonirritant material [13]. 

To date, CoQ10 micelles with a mixture of Kolliphor^®^ HS 15 and stPEG with 40 PEG repeat units have been developed, with the physical properties reported in detailed research [14]. However, to the best of our knowledge, no current literature is available on the preparation and therapeutic effects of micelles consisting only of CoQ10 and stPEG.

In this study, we developed novel CoQ10 micelles using stPEG with different PEG lengths. These micelles, which can be easily prepared using only CoQ10 and stPEG, were evaluated for their properties including drug content, particle size, blood circulation, and in vitro antioxidant ability. Additionally, we assessed the protective effect of this novel formulation against acetaminophen (APAP)-induced acute toxicity in mice to evaluate the in vivo antioxidative efficacy.

## 2. Materials and Methods 

### 2.1. Materials and Animals

CoQ10 was purchased from the Tokyo Chemical Industry (Tokyo, Japan). stPEG10, stPEG25, stPEG40, and stPEG55 were purchased from FUJIFILM Wako Pure Chemical (Osaka, Japan). stPEG140 was kindly supplied by Kao Co., Ltd. (Tokyo, Japan). All other chemicals and reagents were of the highest grades commercially available and used without further purification. Male ddY mice, aged 4 weeks, were purchased from Shimizu Laboratory Supplies (Shizuoka, Japan), housed under standard conditions, and given commercial food and tap water. All animal experiments were conducted in accordance with the institutional guidelines for the care and use of laboratory animals for research, also conforming to the guidelines provided by the Science Council of Japan. The animal studies were approved by the Research Ethics Committee of the Fukuyama University, under registration number H30-animal-7 (approval date: 2018.10.9).

### 2.2. Critical Micelle Concentrations (CMC) Measurements

The CMC of stPEGs were determined by fluorescence spectroscopy (Hitachi 650-10S, Japan) with *N*-phenyl-1-naphthylamine (PNA) as the hydrophobic probe [15]. A known amount of PNA in acetone was individually added to a series of 10-mL vials, and the acetone was evaporated. Next, 2-mL aliquots of stPEG solutions at concentrations from 1.8 to 4000 µg/mL were added to each vial, obtaining a final concentration for PNA of 1 × 10^−7^ M. Here, stPEG10 was prepared in the range of 1.8 to 790 µg/mL as it was dispersed without heating at concentrations above 1000 µg/mL. The sample solutions were incubated at 40 °C for 1 h to allow for the PNA inclusion into the nanoparticles to equilibrate. Next, the samples were cooled at room temperature. The fluorescence emission spectra were scanned between 350 and 600 nm at an excitation wavelength of 340 nm.

### 2.3. Preparation of the CoQ10-Loaded stPEGs Nanoparticles

CoQ10 (40 mg) was placed in a test tube and melted at 60 °C using a water bath. A solution of 200 mg stPEG dissolved in 20 mL distilled water at 60 °C was added to the test tube and sonicated. After centrifuging at 3000 rpm for 5 min, the solution was filtered using a filter paper (Qualitative Filter Paper No.1, ADVANTEC, Japan) and lyophilized. These samples were stored at 4 °C. While using these samples, distilled water or 5% glucose was added and dissolved by sonication.

### 2.4. High-Performance Liquid Chromatography (HPLC) Analysis

HPLC was performed using an HPLC system (LC-20AD, Shimadzu, Kyoto, Japan) equipped with a variable wavelength UV detector (SPD-20A, Shimadzu, Kyoto, Japan). The detection wavelength was 275 nm, and a 4.6 × 150-mm C18 reversed-phase column (TSKgel ODS 80TM, Tosoh, Tokyo, Japan) was used at ambient temperature. The mobile phase was composed of acetonitrile, tetrahydrofuran, and distilled water (55:40:5 (*v/v/v*)), with a flow rate of 1.0 mL/min. The injection volume was 20 μL.

### 2.5. Dynamic Light Scattering (DLS) and Zeta Potential Measurements

DLS was measured as described previously [16]. The zeta potentials of the nanoparticles were determined as follows: approximately 750 µL of each sample solution was placed in a disposable capillary zeta potential cell, and the measurement was performed at 25 °C. The results were the mean values of three experiments performed for each sample.

### 2.6. Saturated Solubility Measurements 

The CoQ10 saturation concentrations of each micelle were measured using the HPLC described above. Briefly, 22.5 mg of CoQ10 equivalent micelles were dissolved in 2 mL of distilled water and centrifuged at 3000 rpm for 30 min. Then, a portion of the aqueous layer was collected and filtered through a 0.45-μm filter, appropriately diluted with the mobile phase to prepare the HPLC samples and injected into the HPLC system as described above.

### 2.7. High-Performance Size-Exclusion Chromatography (HPSEC) Analysis

A three-dimensional analysis was performed using the HPSEC system equipped with liquid chromatography apparatus (LC-10ADVP, Shimadzu, Japan) and a photodiode array detector (Waters 2998, Waters, Massachusetts, United States). A 7.8 × 300-mm TSKgel G4000PWXL column (Tosoh, Japan) was used at 40 °C, where the mobile phase was water, the flow rate was 1.0 mL/min, and the injection volume was 50 μL. The samples were passed through a membrane filter (0.45 μm) before injection.

### 2.8. Blood Circulation

Blood circulation was evaluated as described previously [16] with modifications. Male ddY mice were injected the CoQ10 loaded stPEGs through the tail vein at a dose of 4 mg/kg CoQ10 equivalent in 0.2 mL of 5% glucose. These solutions were filtered using a membrane filter (0.45 μm) before injection. After 1 h, the mice were anesthetized, and blood was collected from the vena cava. The blood was centrifuged at 10,000 rpm for 30 s, and plasma was obtained. These samples were diluted 100 times with a solution of petroleum ether: isopropanol = 4:1, and then left for 1 h at room temperature before centrifuging at 3000 rpm for 5 min. Next, 8 mL of each sample supernatant was pipetted and evaporated at 40 °C. The residue was dissolved in 400 µL of the mobile phase, passed through a membrane filter, and injected into the HPLC system as described above.

### 2.9. In Vitro Antioxidant Ability

The in vitro antioxidative ability was evaluated using the 2,2-diphenyl-1-picrylhydrazyl (DPPH) radical scavenging assay method [17] with slight modifications. In brief, 200 µmol/L of DPPH solution was prepared in 75% ethanol before use and kept away from light. The samples were then dissolved in distilled water to 2 and 4 mg/mL CoQ10 equivalent concentrations. Next, 100 µL was taken from each sample and mixed with 100 µL of DPPH ethanol solution in 96-well plates. The final sample concentrations were 1 and 2 mg/mL CoQ10 equivalent. The mixed solution was incubated for 30 min at 40 °C in the dark and light absorption was measured at 517 nm using Tecan Infinite 200 PRO (TECAN, Switzerland).

A mixture of 100 μL distilled water and 100 μL DPPH solution was used as the “blank 1,” a mixture of 100 μL distilled water and 100 μL 75% ethanol was used as “blank 2,” and a mixture of 100 μL of each sample solution and 100 μL of 75% ethanol was used as a “sample blank.” DPPH radical scavenging activity was evaluated using the following formula.

Radical scavenging activity (%) = ((Absorbance of blank 1 − Absorbance of blank 2) − (Absorbance of sample − Absorbance of sample blank))/(Absorbance of blank 1 − Absorbance of blank 2) × 100.

### 2.10. In Vivo Effects

To evaluate the function of stPEG/CoQ10 micelles in vivo, we measured the protective effect of stPEG55/CoQ10 micelles on acetaminophen (APAP)-induced hepatotoxicity [18] as follows. Mice were administered APAP (400 mg/kg in propylene glycol; intraperitoneally). An intravenous injection of stPEG55/CoQ10 micelles (5 or 15 mg/kg in CoQ10 equivalent) was administered 12 h before the APAP injection. Mice treated with 5% glucose were used as the control group. Six hours after the APAP injection, the mice were anesthetized and blood was collected from the vena cava. The blood was centrifuged at 10,000 rpm for 30 s, and the plasma was obtained. The plasma samples were used to measure aspartate aminotransferase (AST) and alanine aminotransferase (ALT). AST and ALT activities were measured using kits of the POP-TOOS method (FUJIFILM Wako Pure Chemical, Osaka, Japan). Moreover, we measured the protective effect of these micelles on mortality induced by APAP administration. The mice were administered APAP 750 mg/kg intraperitoneally and the stPEG55/CoQ10 micelles were administered as described above. The mice were allowed food and water ad libitum, housed in standard cages, and observed for 28 days. 

### 2.11. Statistical Analysis

Data are expressed as the means ± standard deviation or standard error of the mean. Differences between multiple groups were analyzed using ANOVA followed by Tukey’s multiple comparison test. A value of *p* < 0.05 was considered statistically significant, *p* < 0.01 as highly significant and *p* < 0.001 as extremely significant. Statistical analysis was performed using R, Version 3.5.3 (Vienna, Austria).

## 3. Results

### 3.1. Characterization of the stPEGs and stPEG/CoQ10 Micelles

**Figure 1:**
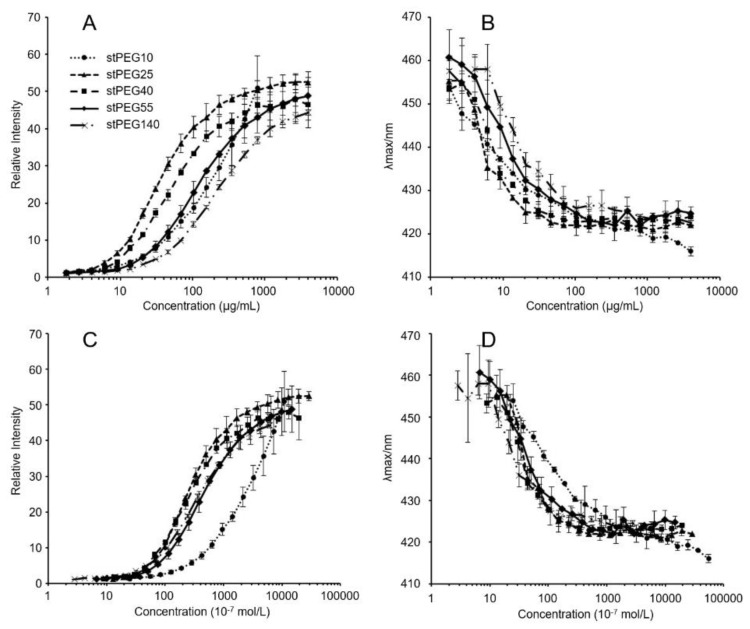
Relative fluorescence intensities (**A**,**C**) and emission maximums (λmax) of PNA as functions (**B**,**D**) of the logarithmic mass (**A**,**B**) or molar (**C**,**D**) concentrations of stPEG10 (●), stPEG25 (▲), stPEG40 (■), stPEG55 (◆), and stPEG140 (×). The values are the means ± S.D. for groups, with the experiment performed in triplicate for each sample.

**Figure 2:**
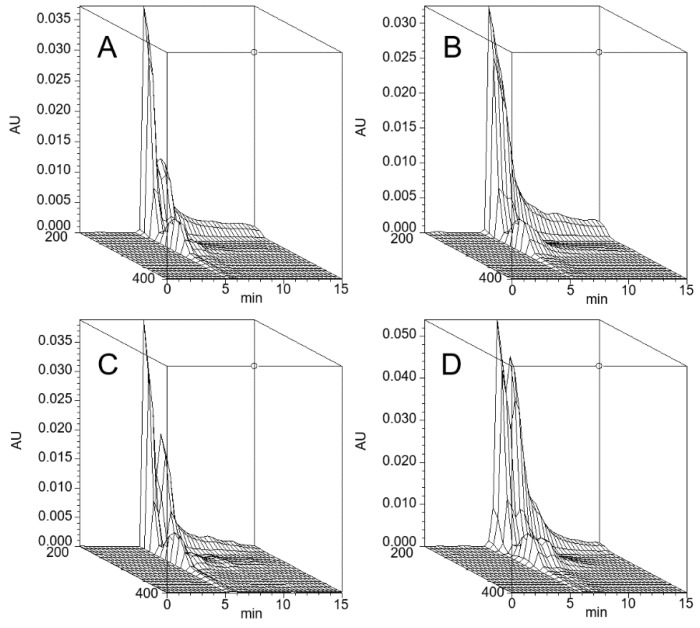
Three-dimensional chromatogram of the stPEG25/CoQ10 micelle (**A**), stPEG40/CoQ10 micelle (**B**), stPEG55/CoQ10 micelle (**C**), and stPEG140/CoQ10 micelle (**D**). High-performance size-exclusion chromatography was performed using an HPLC system equipped with a photodiode array detector. A 7.8 × 300 mm, TSKgel^®^ G4000PWXL column was used at 40 °C. The mobile phase was water, and the flow rate was 1.0 mL/min. AU, Absobance Unit.

The stPEGs formed nanoparticles in an aqueous environment owing to the aggregation of the stearyl group. The CMC of the self-assembled nanoparticles was determined using the fluorescence probe technique with PNA. PNA, which is strongly hydrophobic, has very low water solubility and was preferentially dissolved by the hydrophobic core of the micelles. The relative fluorescence intensity increased with the increasing concentration of each stPEGs, as shown in Figure 1A,C. The curves of the relative fluorescence intensity versus the concentration of each stPEG demonstrated a sharp increase, from which the CMC values were estimated (Table 1) [15]. Furthermore, the shift of the emission maximum of PNA to the lower wavelength, a so-called blue shift, was seen as a concentration function of each stPEG (Figure 1B,D). Mass-based CMC values were the highest for stPEG140. Mole-based CMC values were the highest for stPEG10 and similarly low for other stPEGs. Each stPEG/CoQ10 micellar formulation yielded a clear solution. The size of the unloaded nanoparticles was showed as 44.3 ± 16.8 nm for stPEG10, 34.1 ± 1.8 nm for stPEG140, and approximately 20 nm for other stPEGs, as determined by DLS (Table 1). DLS analysis of the micelles demonstrated that the diameter of the stPEG10/CoQ10 micelle was 58.6 ± 10.9 nm, that of stPEG140/CoQ10 micelle was 32.6 ± 1.6 nm, and other stPEG/CoQ10 micelle diameters were approximately 20 nm. The zeta potentials of these micelles before and after loading CoQ10 were almost equal, and the values were approximately −10 to −25 mV. The CoQ10 content of the stPEG/CoQ10 micelles was determined using HPLC and was approximately 15 *w/w*% (Table 2). The saturation solubility revealed that the stPEG10/CoQ10 micelle had a very low CoQ10 saturation solubility of 0.04 mg/mL; other micelles showed an extremely high CoQ10 saturation solubility of 2.83–9.94 mg/mL (Table 2). The properties of these four stPEG/CoQ10 micelles, with high saturation solubility, were investigated using HPSEC. Figure 2 shows the chromatograms of the stPEG/CoQ10 micelles. The peak retention time of the CoQ10 micelles using stPEG25-55 was 5.6 min and the retention time of stPEG140/CoQ10 micelles was 6.3 min, detected near the excluded volume of the column.

### 3.2. Antioxidant Ability and Blood Circulation of the stPEG/CoQ10 Micelles

**Figure 3:**
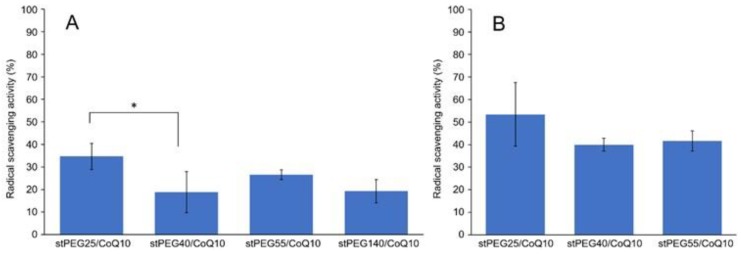
Result of 2,2-diphenyl-1-picrylhydrazyl (DPPH) radical scavenging assay. Inhibition of DPPH radical by stPEG/CoQ10 micelles at 1 mg/mL (**A**) and 2 mg/mL (**B**) as CoQ10 equivalents. The values are the means ± S.D. for groups, with the experiment performed in triplicate for each sample; * *p* < 0.05.

The in vitro antioxidant ability was measured using the DPPH assay method and stPEG25-140/CoQ10 micelles, with confirmed solubility at the experimental concentrations, were assessed. For the stPEG140/CoQ10 micelles, the activity was measured only when the CoQ10 concentration was 1 mg/mL because of the lack of saturation solubility. These micelles demonstrated radical scavenging activity of 18.8–34.7% and 40.0–53.4% at 1 and 2 mg/mL CoQ10 equivalent, respectively. Additionally, all stPEG/CoQ10 micelles demonstrated almost equivalent antioxidant ability (Figure 3). 

**Figure 4:**
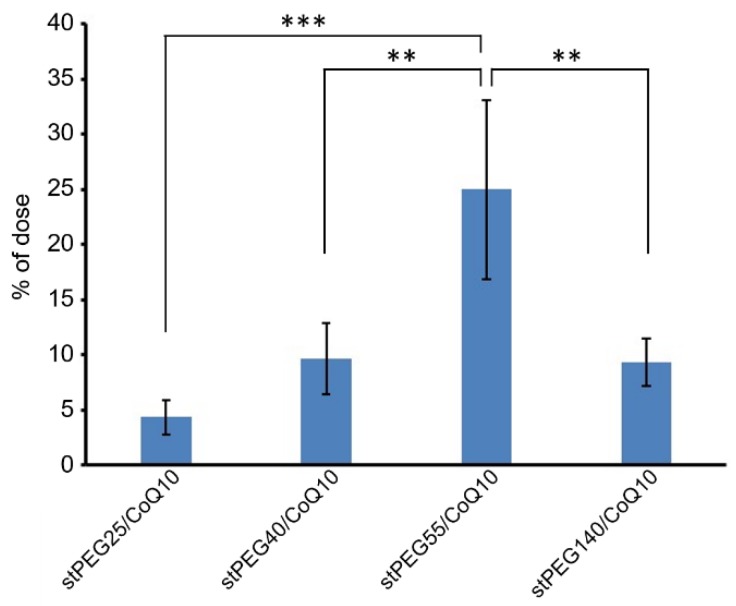
The CoQ10 levels in the plasma 1 h after a single injection of stPEG/CoQ10 micelles with 4 mg/kg in CoQ10 equivalents in mice. The values are the means ± S.D. for groups of four mice; ** *p* < 0.01, *** *p* < 0.001.

**Figure 5:**
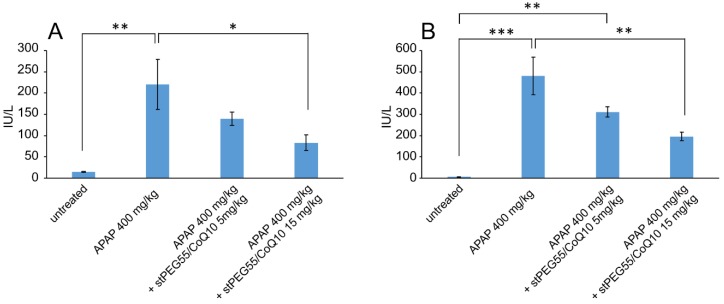
The effect of stPEG55/CoQ10 micelle treatment on aspartate aminotransferase (AST) (**A**) and alanine aminotransferase (ALT) (**B**) levels. Mice were treated with stPEG55/CoQ10 micelle (5, 15 mg/kg CoQ10 equivalent) 12 h before the administration of acetaminophen (APAP) 400 mg/kg. Bar represents means ± S.E., *n* = 4–5 mice per group; * *p* < 0.05, ** *p* < 0.01, *** *p* < 0.001.

Next, the blood circulation of stPEG25-140/CoQ10 micelles was measured using the saturated solubility that can be administered. As shown in Figure 4, the blood circulation after 1 h of administration of stPEG25, 40, 55, 140/CoQ10 micelles was 4.3 ± 1.6%, 9.7 ± 3.2%, 25.0 ± 8.2%, 9.4 ± 2.1% with respect to CoQ10 dose. Furthermore, stPEG55/CoQ10 micelles showed the highest retention in blood. Subsequently, to evaluate the antioxidant ability in vivo, the stPEG55/CoQ10 micelles, demonstrating superior blood circulation of micelles, were used to measure the protective effect against APAP-induced acute toxicity, using AST and ALT as indicators (Figure 5). Following the administration APAP at 400 mg/kg, plasma AST and ALT levels increased significantly. In mice pretreated with stPEG55/CoQ10 micelle, the APAP-induced increased AST and ALT levels were significantly suppressed. This inhibitory effect was dose-dependent for CoQ10.

**Figure 6:**
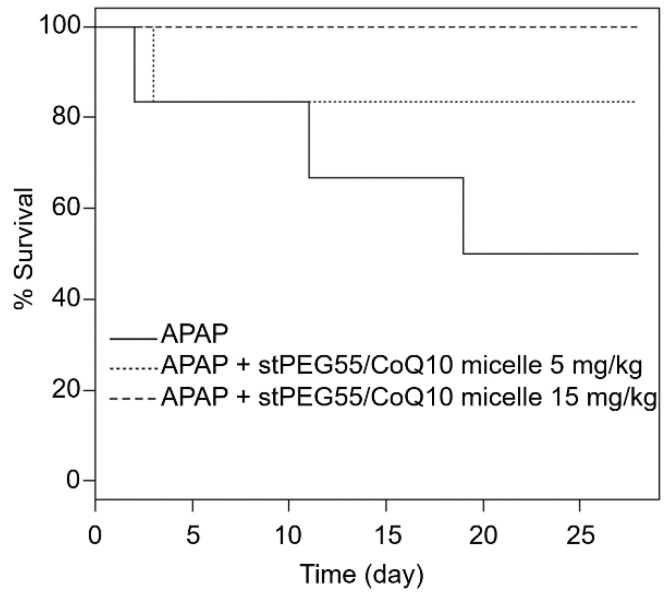
Survival of mice given a lethal dose of APAP; the relationship between stPEG55/CoQ10 micelle administration and effectiveness. Mice were treated with stPEG55/CoQ10 micelle (5, 15 mg/kg CoQ10 equivalent) 12 h before the administration of APAP 750 mg/kg. Six mice were included in each group.

In addition, the effect of stPEG55/CoQ10 micelles was measured using a survival model-administered APAP at 750 mg/kg (Figure 6). The survival rate after 28 days of APAP administration was 50% (3 of 6 mice). However, the survival rate of mice pretreated with 5 mg/kg (CoQ10 equivalent) of stPEG55/CoQ10 micelles was 83% (5 of 6 mice), and the survival rate of mice pretreated with 15 mg/kg (CoQ10 equivalent) of stPEG55/CoQ10 micelles was 100% (6 of 6 mice). Hence, the survival rate was improved in a CoQ10 dose-dependent manner.

## 4. Discussion

This study aimed to develop a new water-soluble CoQ10 formulation that can be administered intravenously. Hence, a micelle formulation using stPEGs was designed. The formation of monodispersive nanoparticles by self-assembly when the stPEGs formed a complex with CoQ10 was studied by using the CMC measurement, DLS, and size-exclusion chromatography. The CMC of the stPEGs was determined by fluorescence spectroscopy with PNA as the hydrophobic probe (Figure 1, Table 1). PNA fluorescence is weak in an aqueous environment but strong in non-polar or hydrophobic environments, such as the inner core of the stPEGs nanoparticles. Notably, the maximum fluorescence emission wavelength shifts toward blue when the PNA molecule moves from an aqueous environment to a hydrophobic one [15]. Hence, we proposed including CoQ10 in the hydrophobic inner core of the nanoparticles. 

Using stPEGs as the amphiphilic block polymer and preparing stPEG solutions at an excessively higher concentration than CMC, we obtained micelle formulations with significantly improved CoQ10 water solubility, except for stPEG10 (Table 2). Reportedly, stPEG is dispersed in water with less than 8 PEG repeat units and dissolved with more than 12 PEG repeat units [13]. The stPEG10/CoQ10 micelle demonstrated extremely poorly soluble in water as stPEG10 may be dispersed in water at high concentrations. This is supported by the fact that, when CMC was measured, stPEG10 was dispersed at a concentration of 1000 µg/mL or higher without heating. Nanoparticle formation and drug loading occurred simultaneously when CoQ10 was heated and sonicated with the aqueous stPEGs solution. Depending on the feed weight ratios of CoQ10 and stPEGs, the amount of CoQ10 loaded into these micelles was approximately 15 *w/w*% (Table 2). In addition, the loaded CoQ10 demonstrated a dose-dependent antioxidant ability; this effect was almost unaffected by the type of stPEG (Figure 3). As shown in Table 1; Table 2, DLS measurement indicated that drug loading in the inner core did not affect the size of the micelles. The diameter of stPEG/CoQ10 micelles was approximately 15–60 nm (Table 2). Nanoparticles less than 100 nm in diameter have been reported to increase the blood circulation to avoid rapid trapping by the reticuloendothelial system of the liver, spleen, and lung [19]. The PEGylation of the nanoparticle surface significantly improves the circulation time [20]. Furthermore, as shown in Figure 2, these micelles were detected near the excluded volume of the column in HPSEC analysis, suggesting that stPEG/CoQ10 micelles have a stable structure. The negative zeta potential is also thought to contribute to the stability of the nanostructure.

Based on the above rationale, stPEG/CoQ10 micelles were expected to have a long circulation time. Therefore, we evaluated the blood circulation of these micelles (Figure 4). Among the evaluated micelles, the stPEG55/CoQ10 micelle showed the highest blood circulation, with approximately 25% of the dose of CoQ10 was present in plasma 1 h after administration. However, the blood circulation of other micelles was relatively low, indicating that the blood circulation cannot be solely predicted based on particle size, zeta potential, and stability. Furthermore, there seems to be an optimal PEG length crucial for the blood circulation of micelles. Finally, the protective ability of stPEG/CoQ10 micelles in APAP-induced acute liver injury mice was evaluated based on biochemical tests and survival rates using the stPEG55/CoQ10 micelle as a representative example (Figure 5 and Figure 6). Both biochemical tests and survival rates were shown to improve with CoQ10, in a dose-dependent manner. For example, the intravenous administration of APAP is a very safe drug used for post-operative pain control, but older patients demonstrated hepatotoxicity even when administered within the normal dose range [21]. In patients with such characteristics, pre-administration of the stPEG/CoQ10 micellar formulation prior to APAP administration might prevent liver damage. 

In this study, APAP was used as a drug-inducing oxidative stress, and it was indicated that this micelle reduces the side effects of oxidative stress. In addition to APAP, many drugs cause side effects because of oxidative stress, such as anthracycline-induced cardiotoxicity [22]. Hence, this micelle formulation can be effectively used to treat the side effects of these oxidative stress-inducing drugs. Furthermore, CoQ10 has been shown to be effective against cardiovascular diseases, different cancers, reproductive disorders, mitochondrial diseases, and Parkinson’s disease [23,24,25,26], suggesting that the novel micellar formulation developed in this study might find application in these diseases.

## 5. Conclusions

In this study, the physical properties of five kinds of PEG length stPEG were evaluated. In addition, the preparation method, physical properties, blood circulation, and drug efficacy of stPEG/CoQ10 micelles were evaluated. CoQ10 micelles prepared with stPEG25-55, with a high saturation solubility, demonstrated a similar radical scavenging ability in vitro. Additionally, of the stPEGs evaluated in this study, the CoQ10 micelle consisting of 55 PEG units demonstrated a significantly higher blood circulation than the other micelles. The stPEG55/CoQ10 micelle was shown to have protective effects in an oxidative stress mouse model. Based on the above, it is expected that stPEG55/CoQ10 micelles could be an inexpensive injectable preparation with high solubility. In the future, as part of further investigations into the safety and efficacy of this CoQ10 micelle, it is crucial to evaluate detailed blood circulation and organ distribution with time sequence, and therapeutic effects in other oxidative stress models, including anthracycline-induced cardiotoxicity.

## Figures and Tables

**Table 1:** Properties of the polyethylene glycol monostearate (stPEGs).

Sample	Molecular Weight	CMC ^†^ (µg/mL)	CMC ^†^ (µmol/L)	Diameter (nm) ^†^	Zeta Potential (mV) ^‡^
stPEG10	725.0	9.4	13.00	44.3 ± 16.8	−22.2 ± 1.5
stPEG25	1385.8	4.5	3.25	19.2 ± 3.5	−10.4 ± 2.9
stPEG40	2046.3	6.7	3.25	17.2 ± 1.6	−15.2 ± 4.0
stPEG55	2707.4	7.0	2.60	19.7 ± 3.8	−15.9 ± 0.4
stPEG140	6451.9	10.2	15.8	34.1 ± 1.8	−14.9 ± 1.2

^†^ CMC, Critical Micelle Concentrations. ^‡^ Values are expressed as the means ± standard deviation (n = 3).

**Table 2:** Properties of the stPEG/CoQ10 micelles.

Sample	CoQ10 Content (*w/w*%)	Diameter (nm)	Zeta Potential (mV)	Saturated Solubility (mg/mL) ^†^
stPEG10/CoQ10 micelle	13.7 ± 2.6	58.6 ± 10.9	−23.9 ± 17.1	0.04 ± 0.01
stPEG25/CoQ10 micelle	14.9 ± 0.9	18.5 ± 0.6	−10.9 ± 7.5	9.94 ± 0.46
stPEG40/CoQ10 micelle	14.6 ± 1.7	17.7 ± 2.6	−15.8 ± 3.0	7.38 ± 0.06
stPEG55/CoQ10 micelle	15.5 ± 2.0	22.1 ± 2.1	−18.9 ± 6.1	9.11 ± 0.24
stPEG140/CoQ10 micelle	18.1 ± 3.3	32.6 ± 1.6	−20.6 ± 9.8	2.83 ± 0.41

Values are expressed as the means ± standard deviation (*n* = 3). ^†^ Saturated solubility is expressed as CoQ10 equivalents.
